# Supplementary material for: The causal role of circulating inflammatory markers in osteoporosis: a bidirectional Mendelian randomized study
Source: Front Immunol. 2024 Jul 18;15:1412298. doi: 10.3389/fimmu.2024.1412298 (PMC11291241; doi:10.3389/fimmu.2024.1412298)
Supplement: Supplementary file 1 [file DataSheet_1.zip › supplementary material/OSTEOPOROSIS-CIM/GCST90274762.tsv.gz_forest.pdf]

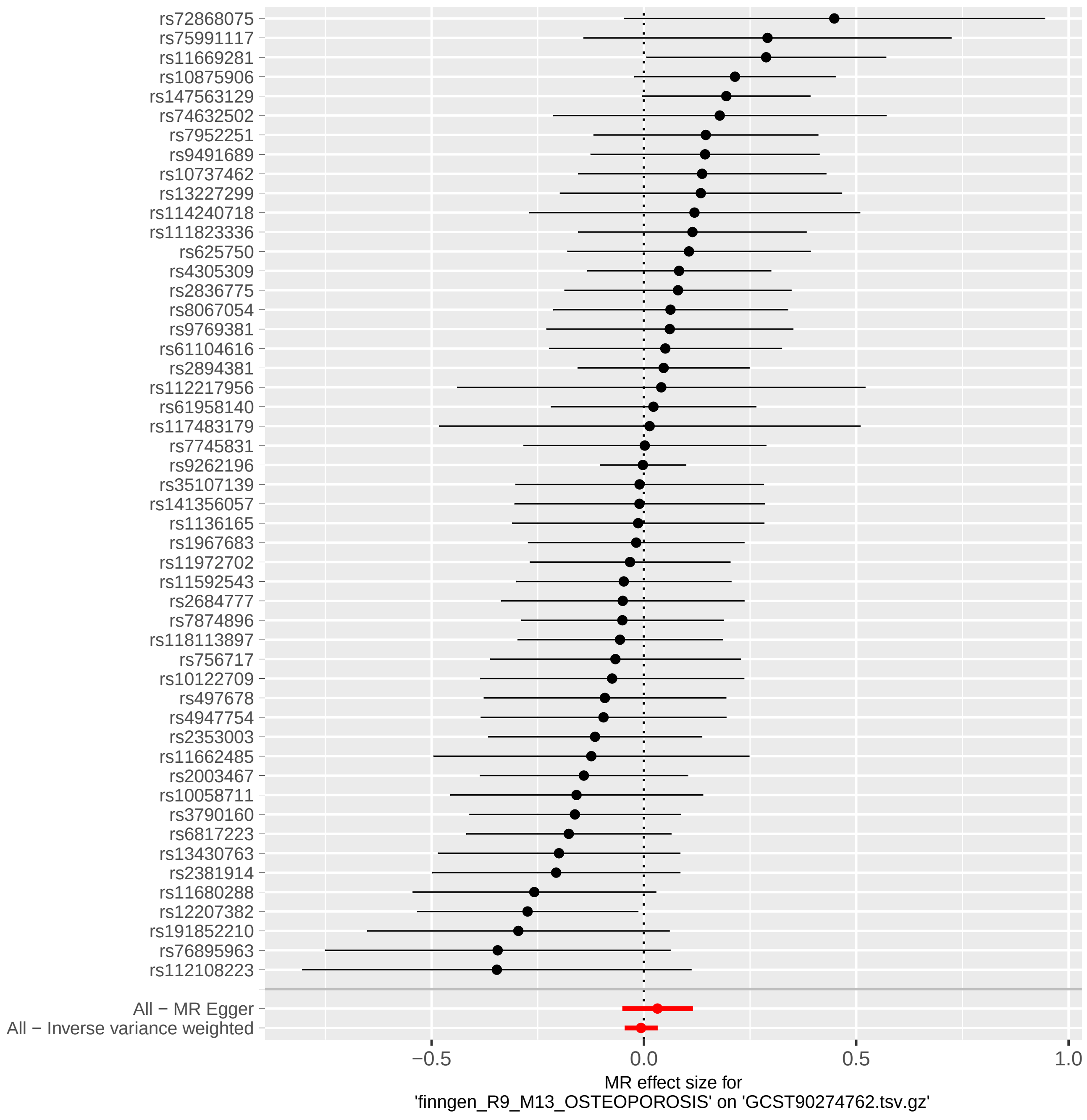

rs72868075
rs75991117
rs11669281
rs10875906
rs147563129
rs74632502
rs7952251
rs9491689
rs10737462
rs13227299
rs114240718
rs111823336
rs625750
rs4305309
rs2836775
rs8067054
rs9769381
rs61104616
rs2894381
rs112217956
rs61958140
rs117483179
rs7745831
rs9262196
rs35107139
rs141356057
rs1136165
rs1967683
rs11972702
rs11592543
rs2684777
rs7874896
rs118113897
rs756717
rs10122709
rs497678
rs4947754
rs2353003
rs11662485
rs2003467
rs10058711
rs3790160
rs6817223
rs13430763
rs2381914
rs11680288
rs12207382
rs191852210
rs76895963
rs112108223
All − MR Egger
All − Inverse variance weighted
−0.5
0.0
0.5
1.0
MR effect size for
'finngen_R9_M13_OSTEOPOROSIS' on 'GCST90274762.tsv.gz'
